# Supplementary material for: An enhancer cluster controls gene activity and topology of the SCN5A-SCN10A locus in vivo
Source: Nat Commun. 2019 Oct 30;10:4943. doi: 10.1038/s41467-019-12856-5 (PMC6821807; doi:10.1038/s41467-019-12856-5)
Supplement: Supplementary file 2 — Supplementary Information [file 41467_2019_12856_MOESM2_ESM.pdf]

An enhancer cluster controls gene activity and topology of the SCN5A-SCN10A locus in vivo

Man et al.

Supplementary Information

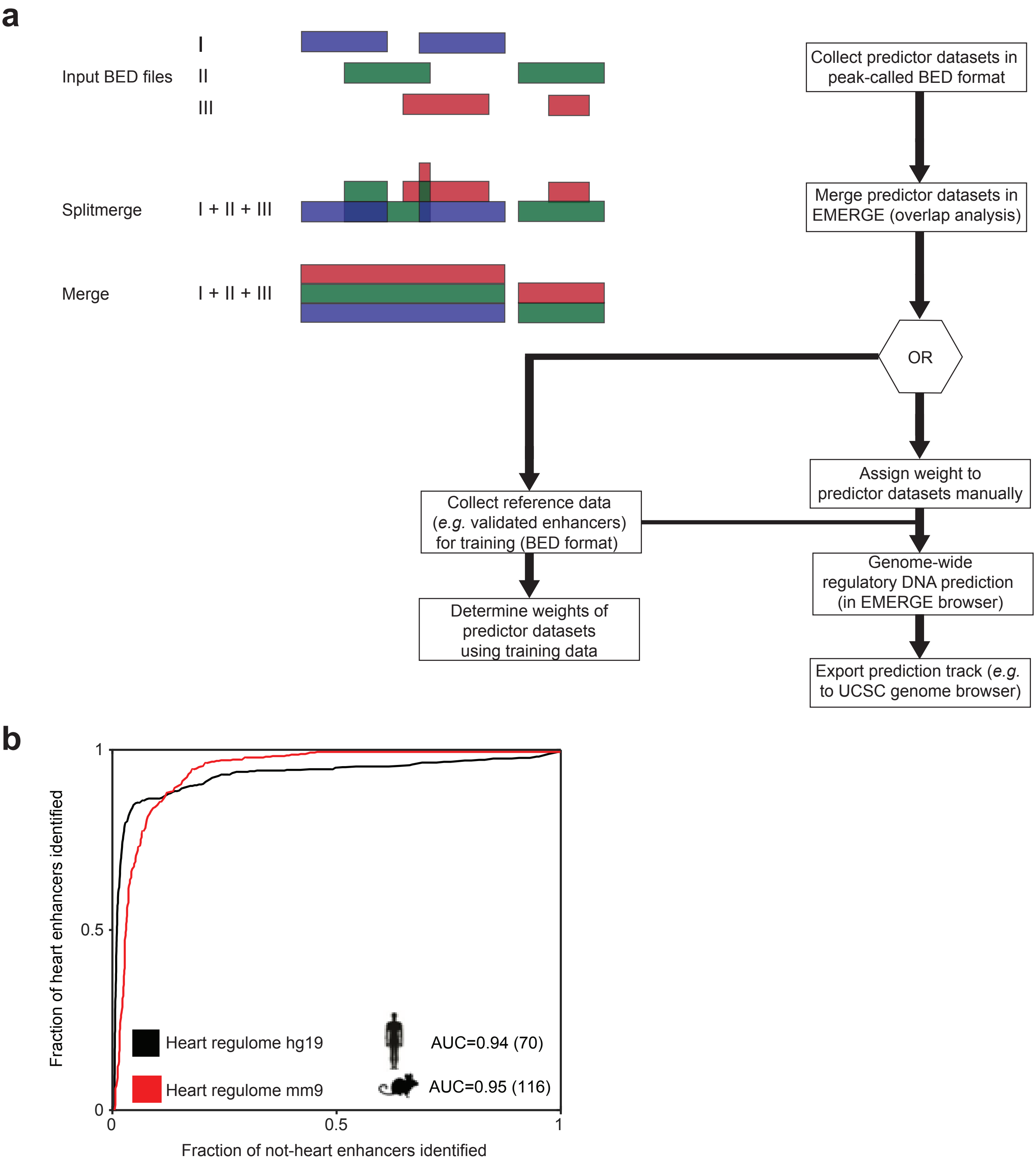

Supplementary Figure 1. Overview of the EMERGE framework. **a** The EMERGE flowchart, including the collecting and merging of input BED files, the assignment of dataset weights for prediction and the possibility to export the resulting prediction tracks. **b** Plotted Receiver operating characteristic (ROC) curves belonging to the mouse and human heart regulome catalogues predicted using EMERGE. Area under the curve (AUC) values are given for each ROC curve. The number of functional genomic predictor datasets used for the predictions is given in brackets. Predictions were generated using a subset of a set of highly active human and mouse heart validated heart enhancers (n=84 for both) from the VISTA enhancer repository[1] to train EMERGE. Different subsets of the same enhancer sets were subsequently sub-sampled for testing to construct ROC curves, as described in the original EMERGE paper[2].

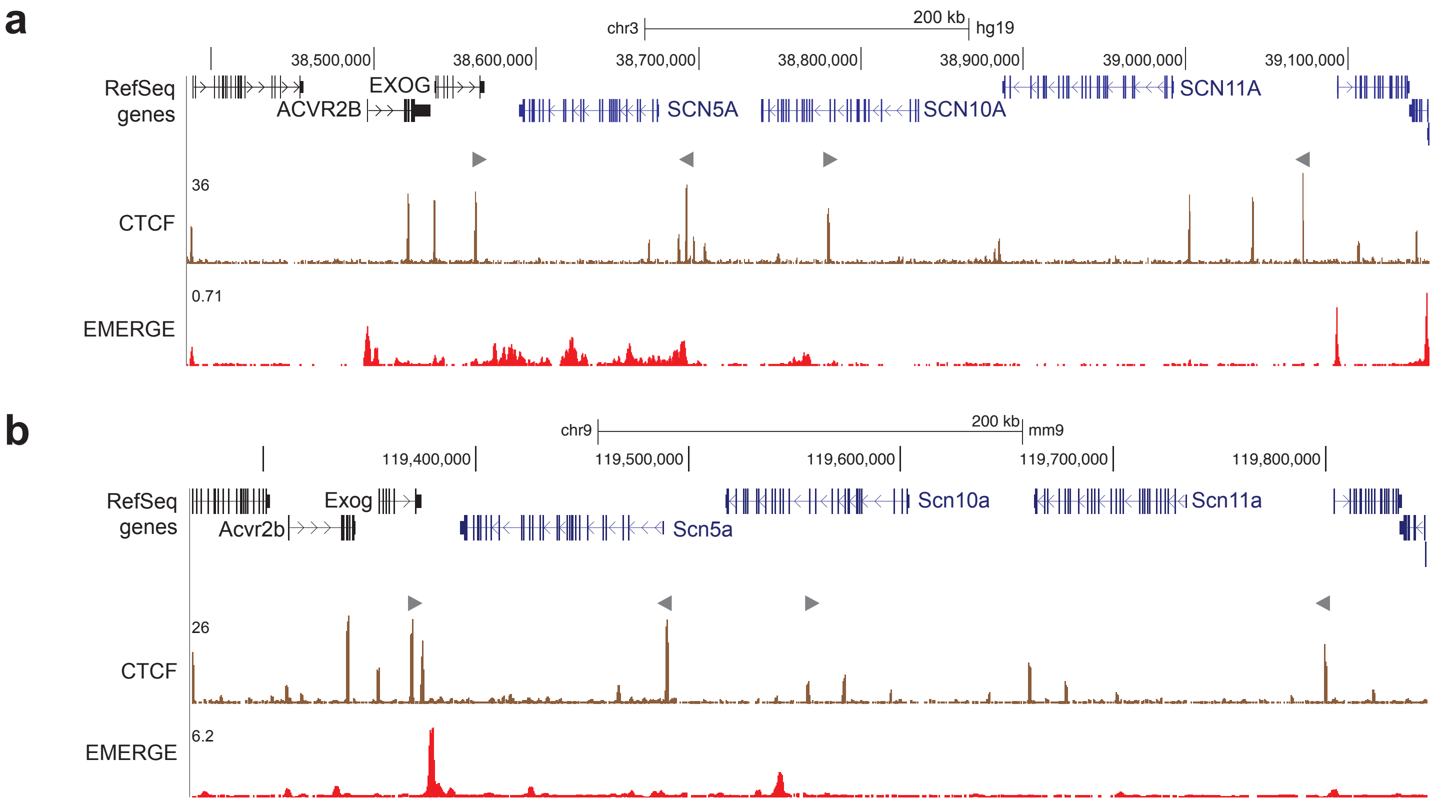

Supplementary Figure 2. CTCF-mediated chromatin loops in the human *SCN5A-SCN10A* locus and mouse *Scn5a-Scn10a* locus. **a**, **b** Human S*CN5A-SCN10A* locus (**a**) and mouse *Scn5a-Scn10a* locus (**b**) showing the forward and reverse orientation of conserved CTCF sites (grey triangles). The *SCN5A/Scn5a* TAD is confined between convergent CTCF sites. RE6-9 and the *SCN5A/Scn5a* promoter are flanked by convergent CTCF sites. Underneath human and mouse EMERGE.

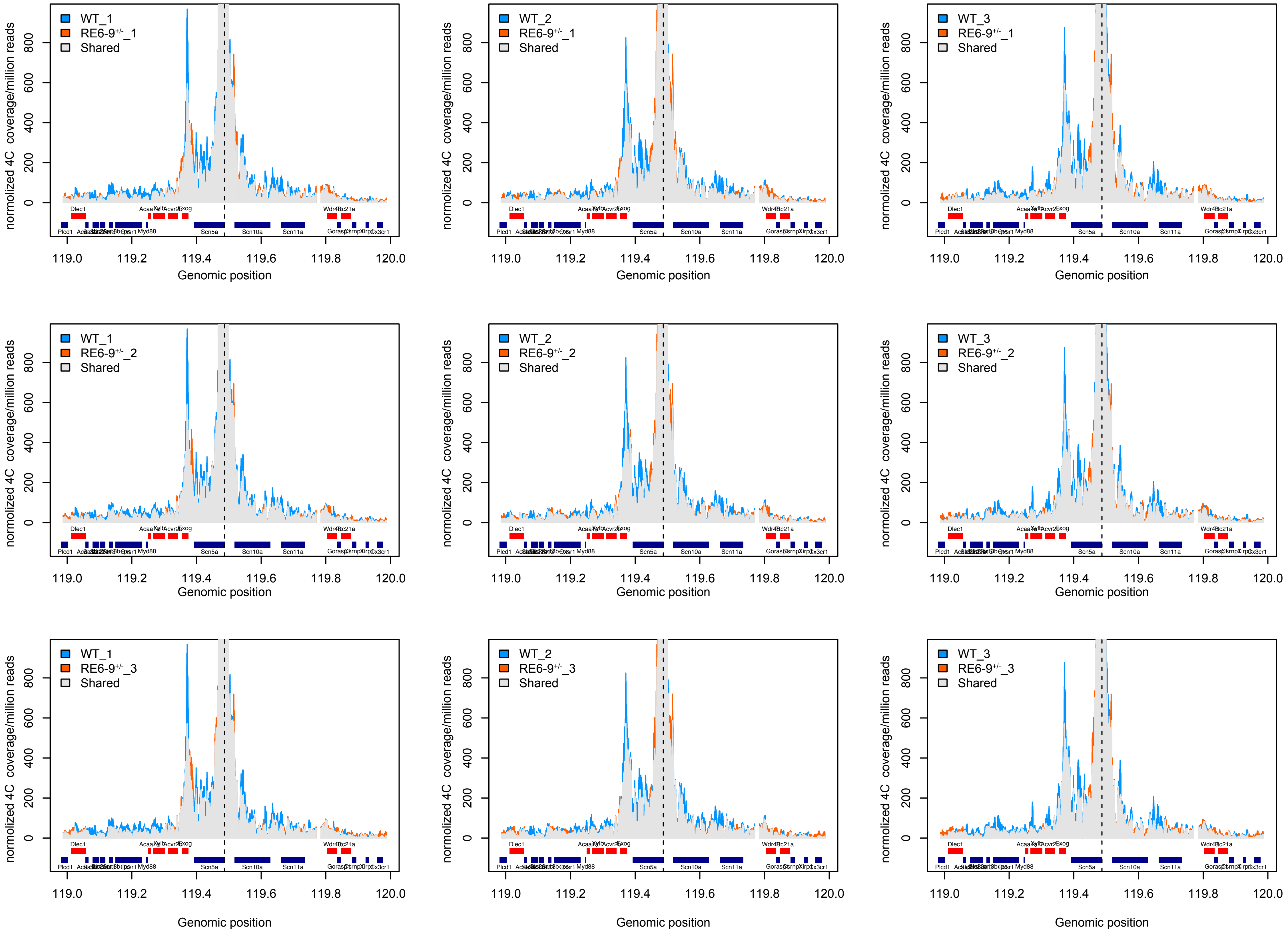

Supplementary Figure 3. Overlay of 4C contact profiles per replicate from viewpoint *Scn5a* promoter. 4C contact profiles showing the overlay for each combination per RE6-9$^{+/-}$ replicate (n=3) with wildtype (n=3). Upper panel = RE6-9$^{+/-}$_1; middle panel = RE6-9$^{+/-}$_2; bottom panel = RE6-9$^{+/-}$_3. Blue signal = WT; orange signal = RE6-9$^{+/-}$; grey signal = shared.

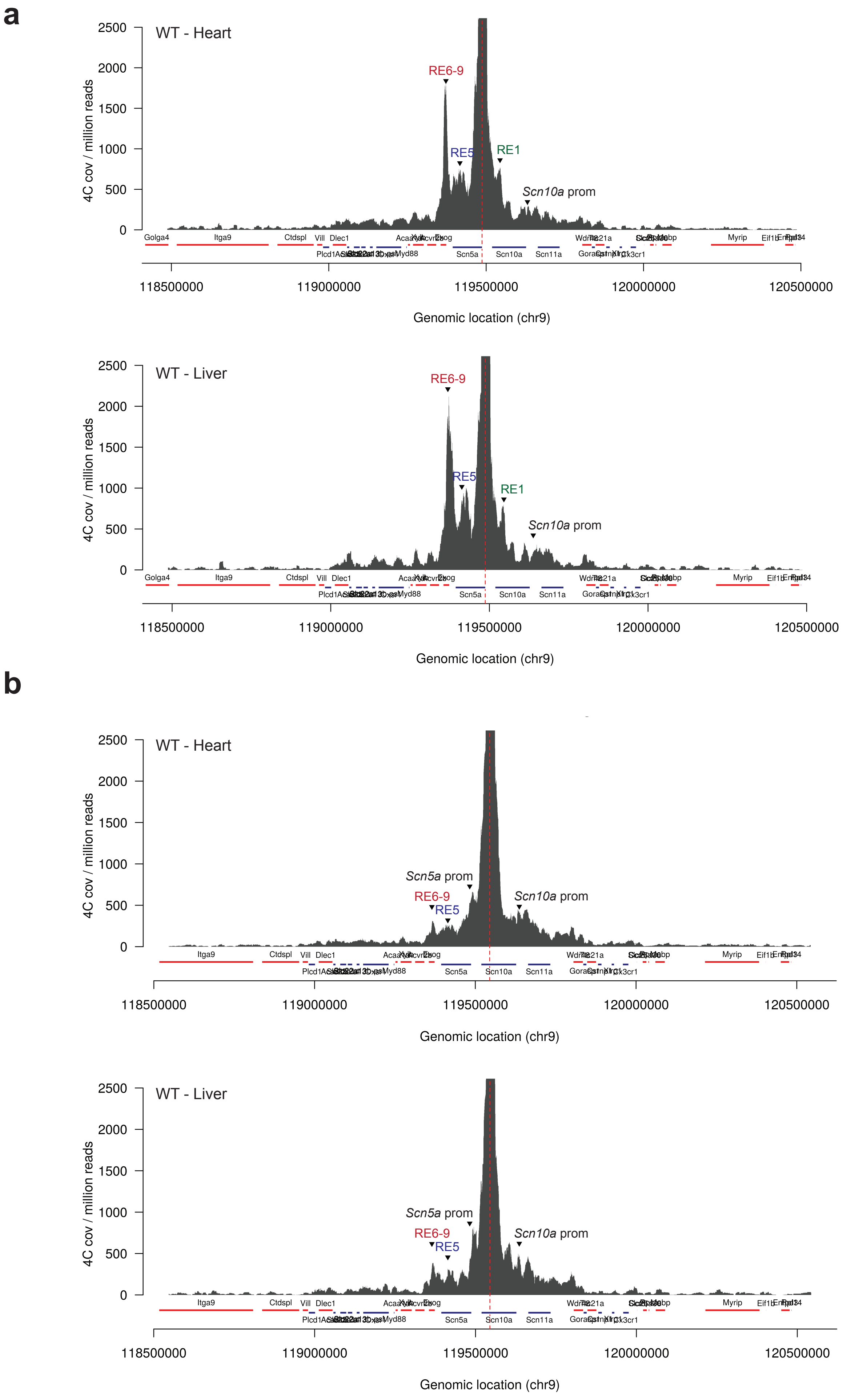

Supplementary Figure 4. The three-dimensional organization of the *Scn5a-Scn10a* in liver. **a, b** Shown a typical example of 4C contact profiles of wildtype heart (n=3) versus liver (n=3) for *Scn5a* promoter (**a**) viewpoint and RE1 viewpoint (**b**).

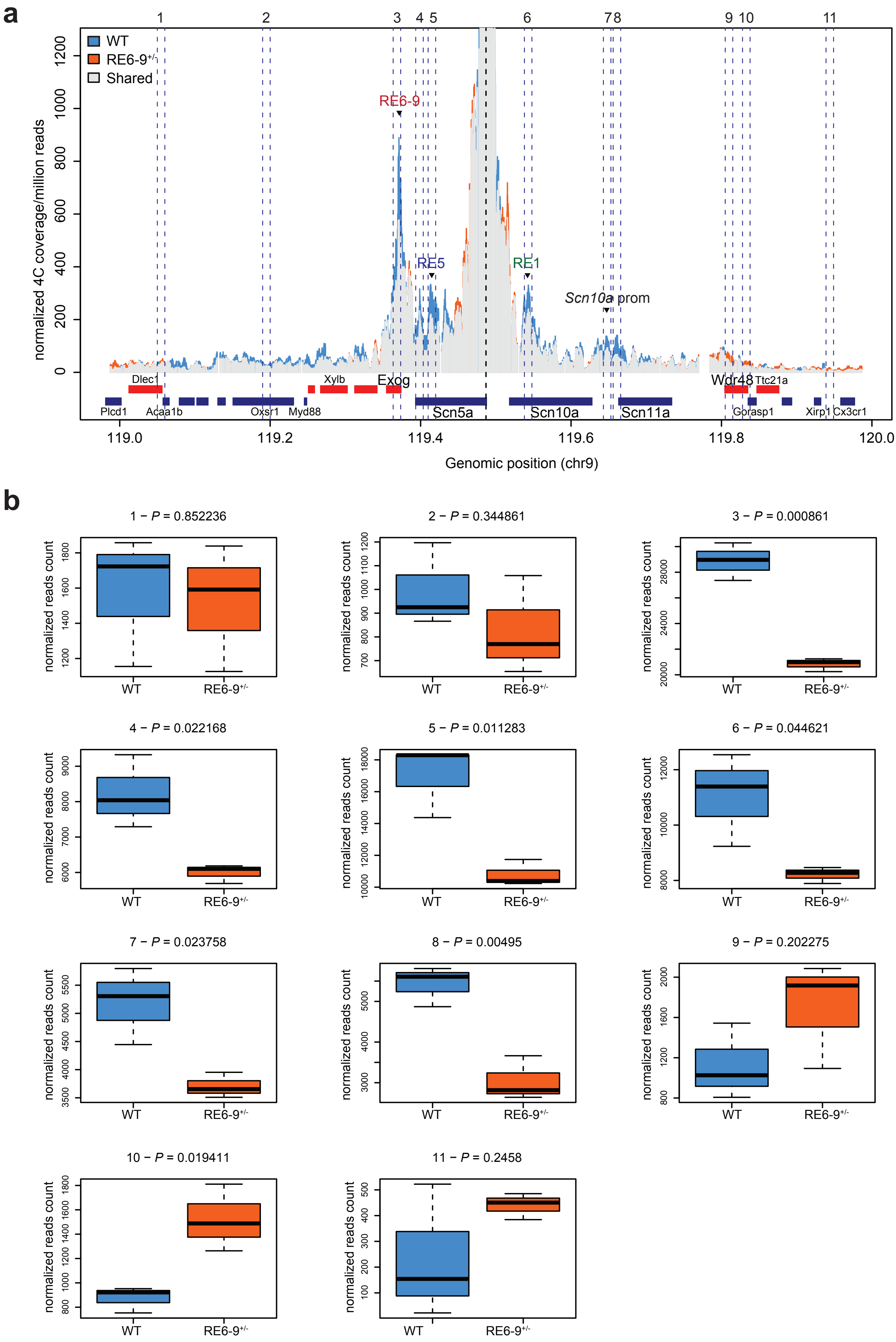

Supplementary Figure 5. Statistical analysis of 4C contact profiles from viewpoint bait *Scn5a* promoter. **a** Genomic locations in the *Scn5a*-*Scn10a* tested for difference in mean in contact profiles: 1. 119055000, 2. 119195000, 3. 119368500, 4. 119398500, 5. 119415000, 6. 119543000, 7. 119648000, 8. 119661000, 9. 119810000, 10. 119833000, 11. 119944000. **b** Boxplots showing the difference in normalized reads count per measured anchor. *P* values were calculated using unpaired Student's *t*-test. 3 = RE6-9; 5 = RE5; 6 = RE1; 7/8 = *Scn10a* promoter.

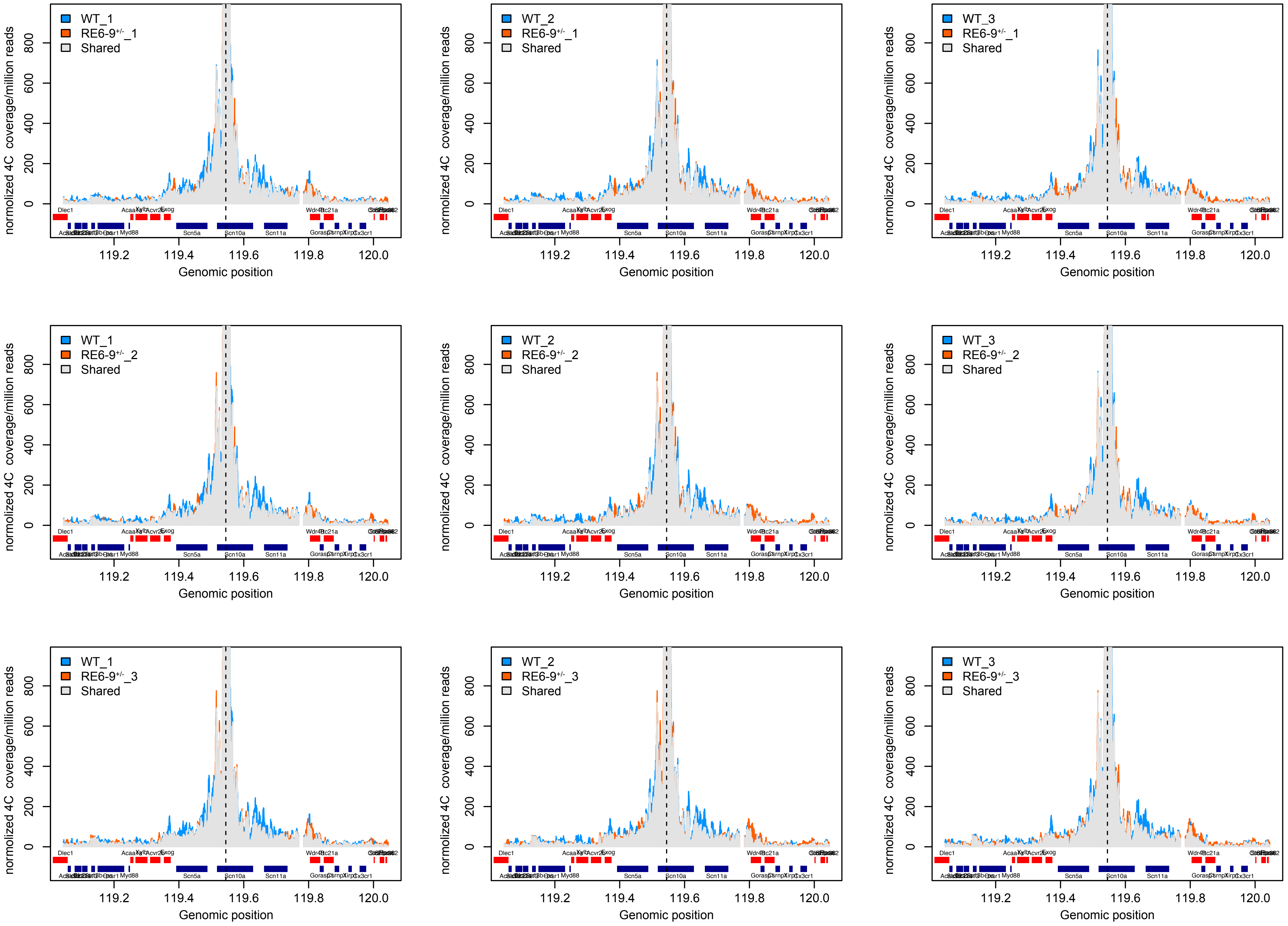

Supplementary Figure 6. Overlay of 4C contact profiles per replicate from viewpoint RE1. 4C contact profiles showing the overlay for each combination per RE6-9$^{+/-}$ replicate (n=3) with wildtype (n=3). Upper panel = RE6-9$^{+/-}$_1; middle panel = RE6-9$^{+/-}$_2; bottom panel = RE6-9$^{+/-}$_3. Blue signal = WT; orange signal = RE6-9$^{+/-}$; grey signal = shared.

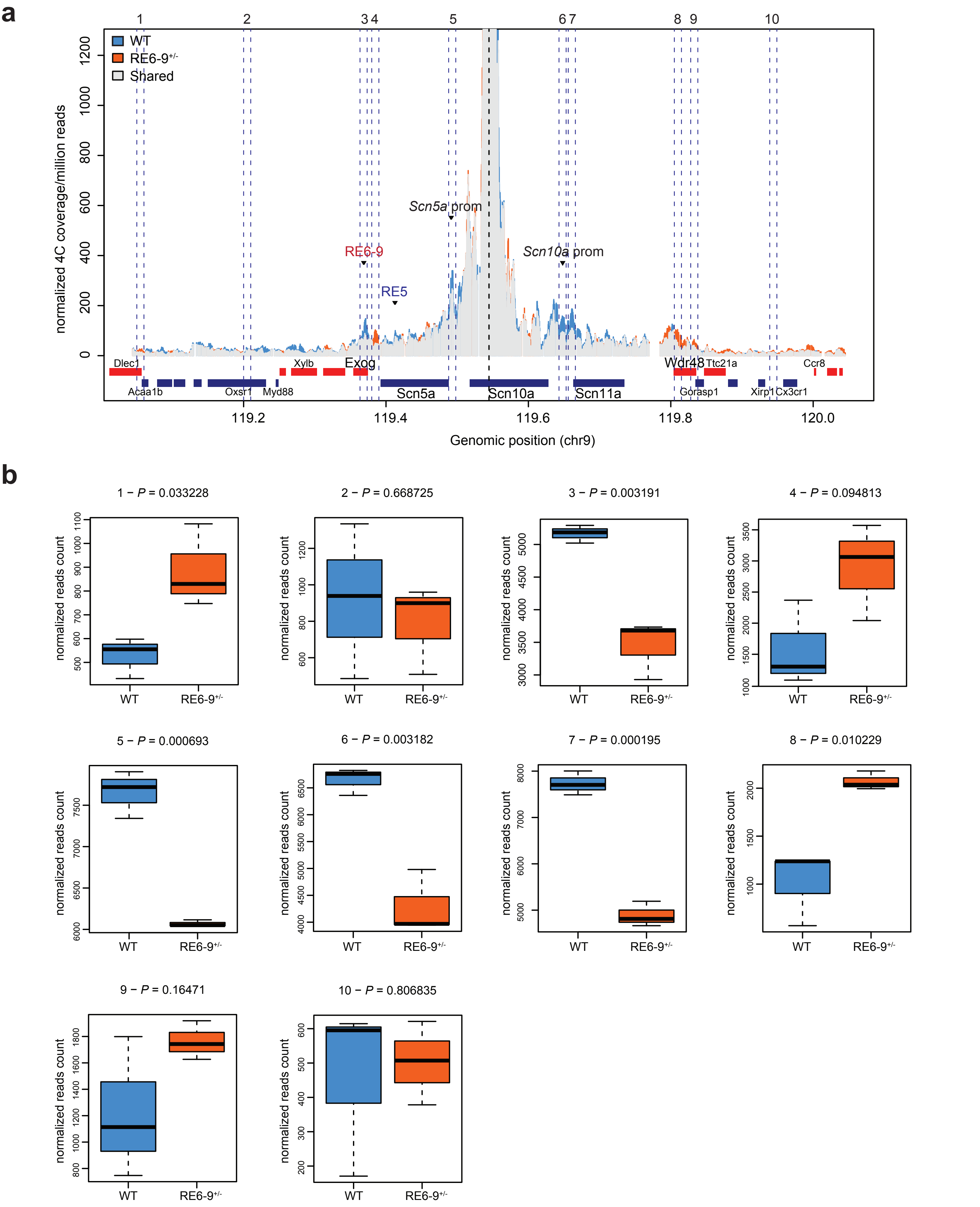

Supplementary Figure 7. Statistical analysis of 4C contact profiles from viewpoint bait RE1. **a** Genomic locations in the *Scn5a*-*Scn10a* tested for difference in mean in contact profiles: 1. 119055000, 2. 119205000, 3. 119368500, 4. 119385000, 5. 119493000, 6. 119648000, 7. 119661000, 8. 119810000, 9. 119833000, 10. 119944000. **b** Boxplots showing the difference in normalized reads count per measured anchor. *P* values were calculated using unpaired Student's *t*-test. 3 = RE6-9; 5 = *Scn5a* promoter; 6/7 = *Scn10a* promoter.

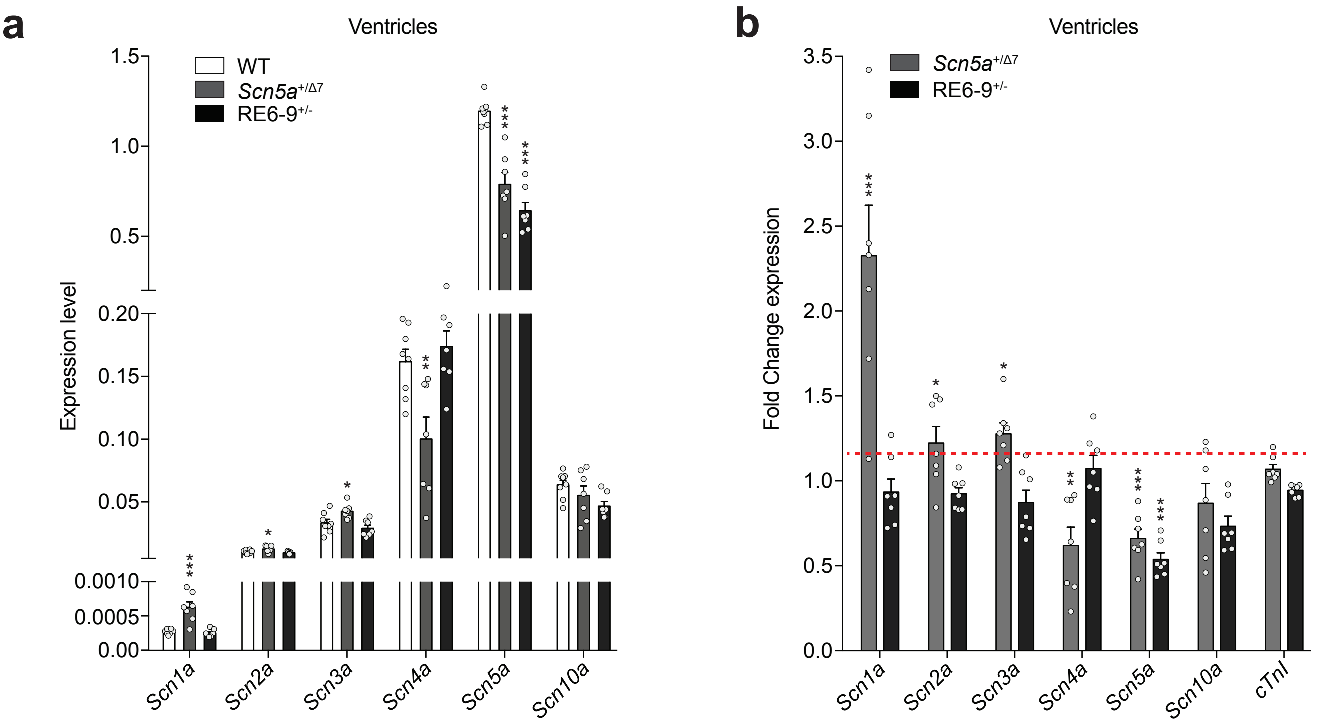

Supplementary Figure 8. Transcriptional adaption of several sodium channel gene family members in heterozygous *Scn5a*$^{+/\Delta7}$ mice. **a, b** Absolute expression (**a**) and fold change expression (**b**) levels of *Scn1a*, *Scn2a*, *Scn3a*, *Scn4a*, *Scn5a* and *Scn10a* in ventricles from 8-week old *Scn5a*$^{+/\Delta7}$ and RE6-9$^{+/-}$ mice compared to wildtype (WT). Bars represent mean ± SEM and *P* values were calculated using one-way ANOVA with post-hoc Bonferroni tests. WT n=8; *Scn5a*$^{+/\Delta7}$ n=7; RE6-9$^{+/-}$ n=7. **P* < 0.05; ***P* < 0.01; ****P* < 0.001. Source data are provided as a Source Data file.

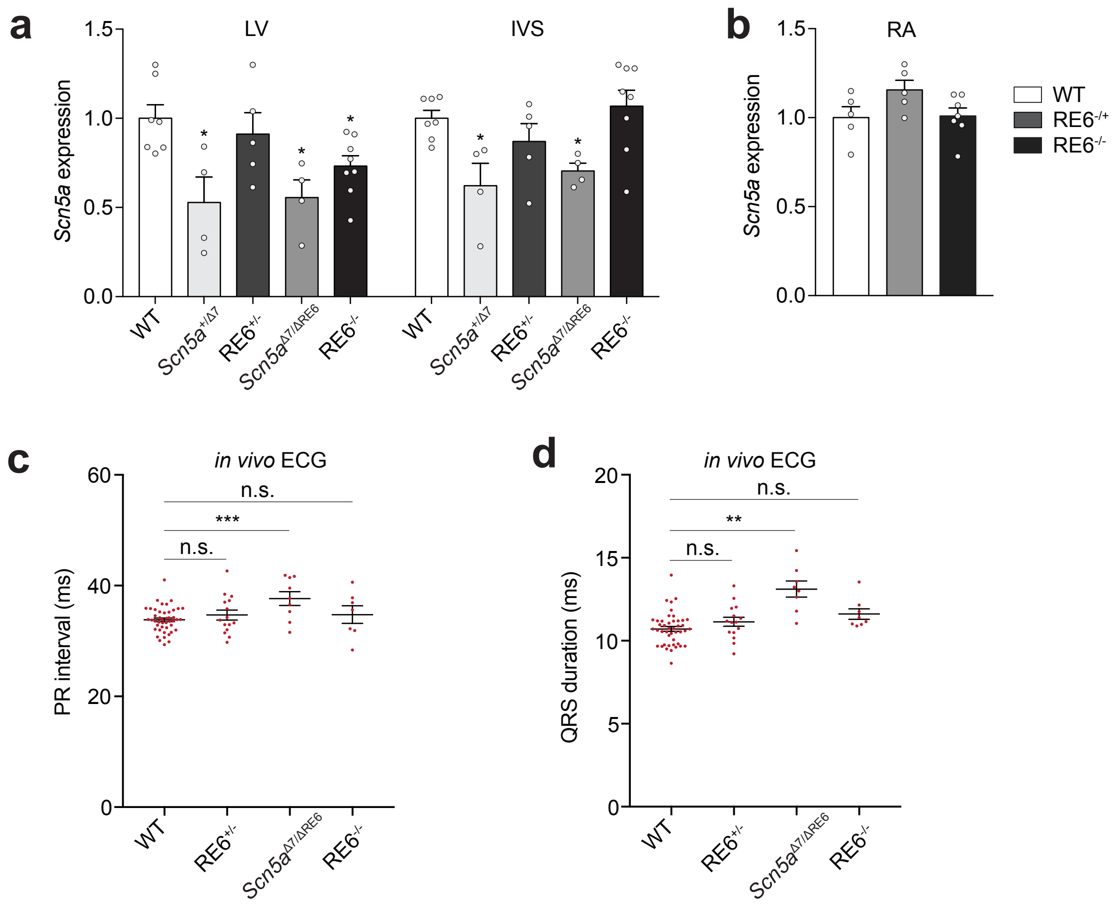

Supplementary Figure 9. Regulatory function of RE6 is minimal in *Scn5a* expression and function in the heart. **a** Fold change expression level of *Scn5a* from left ventricle and interventricular septum, and **b** right atrium of *Scn5a*$^{+/\Delta7}$, RE6$^{+/-}$, RE6$^{-/-}$ and compound heterozygous *Scn5a*$^{\Delta7/\Delta RE6}$ mutants. *P* values were calculated using one-way ANOVA with post-hoc Bonferroni tests. WT n=4; *Scn5a*$^{+/\Delta7}$ n=3; RE6$^{+/-}$ n=5; *Scn5a*$^{\Delta7/\Delta RE6}$ n=4; RE6$^{+/-}$ n=8. *$P < 0.05$. **c, d** *in vivo* ECG analysis of RE6$^{+/-}$ and RE6$^{-/-}$ showing PR interval and QRS duration. *P* values were calculated using one-way ANOVA with post-hoc Bonferroni tests. WT n=44; RE6$^{+/-}$ n=15; *Scn5a*$^{\Delta7/\Delta RE6}$ n=8; RE6$^{-/-}$ n=8. **$P < 0.01$; **$P < 0.001$; Bars represent mean ± SEM; n.s. not significant. LV = left ventricle; IVS = interventricular septum; RA = right atrium. Source data are provided as a Source Data file.

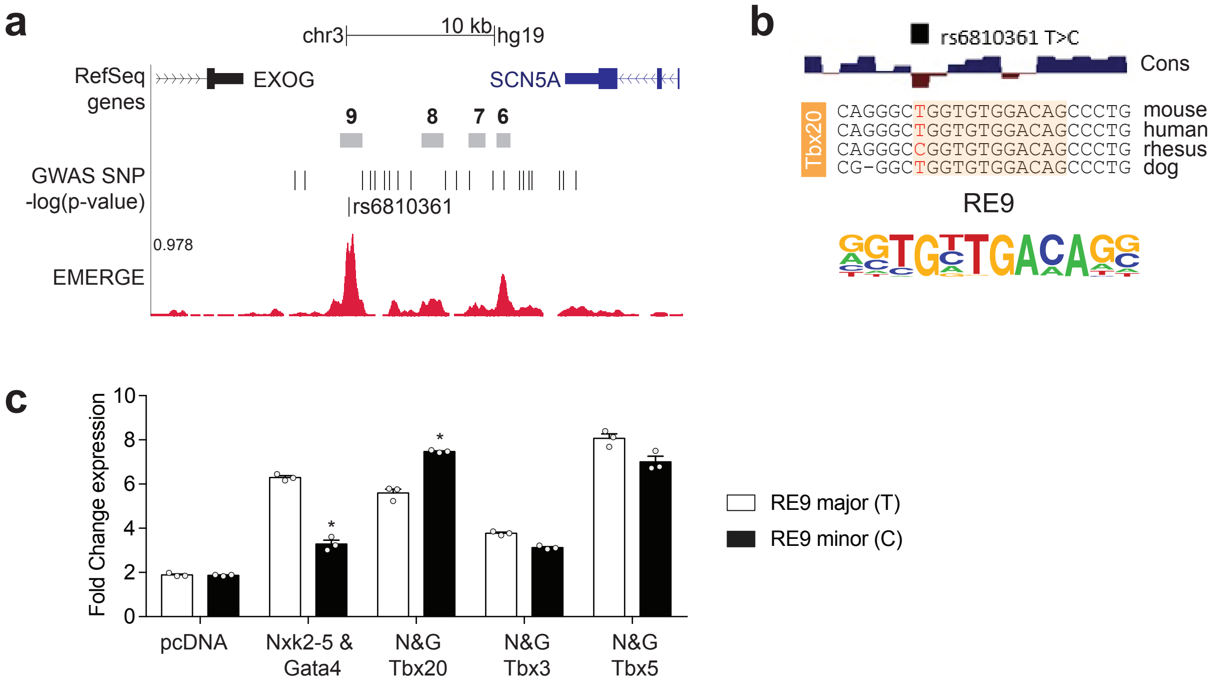

Supplementary Figure 10. A genetic variant affecting the activity of the enhancer cluster. **a** UCSC browser view of the enhancer cluster RE6-9 including QRS-associated genetic variant rs6810361 (T>C) positioned in RE9 and EMERGE. **b** rs6810361 (T>C, red) is located at the edge of a conserved Tbx20 binding site including sequence alignment between species and JASPAR consensus site **c** Luciferase activity assay in H10 cells. Addition of cardiac transcription factors Nkx2-5 and Gata4 causes strong reduction in RE9 activity for the minor allele. This effect is possibly rescued by Tbx20. Bars represent mean ± SEM and *P* values were calculated using two-way ANOVA with post-hoc Bonferroni tests. Replicates n=3.*$P < 0.05$. Source data are provided as a Source Data file.

Dataset	Type	Genome	Tissue	Overall	Stage	Source	Deposit	# of Peaks
Conservation	PhastCons	mm9	all	all	all	Siepel lab	UCSC	972010
CTCF	ChIP-seq	mm9	heart	heart	adult	Ren lab	GSM918756	41866
DHSs	Dnase1 hypersensitivity	mm9	whole_brain	brain	E18.5	ENCODE	GSE37074	143557
H3K27ac	ChIP-seq	mm9	whole_brain	brain	E14.5	ENCODE	GSE49847	34012
H3K4me1	ChIP-seq	mm9	whole_brain	brain	E14.5	ENCODE	GSE49847	152915
H3K4me3	ChIP-seq	mm9	whole_brain	brain	E14.5	ENCODE	GSE49847	36227
Pol2	ChIP-seq	mm9	whole_brain	brain	E14.5	ENCODE	GSE49847	17146
Smarca4	ChIP-seq	mm9	forebraim	brain	E11.5	Pennacchio lab	GSE37151	7349
ATAC	ATAC-seq	mm9	Ventricle	heart	E12.5	Christoffels lab	in house	84667
ATAC	ATAC-seq	mm9	right arium	heart	E12.5	Christoffels lab	in house	129996
ATAC	ATAC-seq	mm9	left atrium	heart	E12.5	Christoffels lab	in house	125461
ATAC	ATAC-seq	mm9	HL-1 cells	heart	no stimulation 20k	Christoffels lab	in house	110206
ATAC	ATAC-seq	mm9	HL-1 cells	heart	no stimulation 50k	Christoffels lab	in house	150324
ATAC	ATAC-seq	mm9	HL-1 cells	heart	Bmp2 stim 20k	Christoffels lab	in house	150279
ATAC	ATAC-seq	mm9	HL-1 cells	heart	Bmp2 stim 50k	Christoffels lab	in house	130868
ATAC	ATAC-seq	mm9	atria	heart	Embryonic	Moskowitz	unpublished	18213
ATAC	ATAC-seq	mm9	pSHF	heart	Embryonic	Moskowitz	unpublished	21318
ATAC	ATAC-seq	mm9	ventricles	heart	Embryonic	Moskowitz	unpublished	33418
Coup-TFII	ChIP-seq	mm9	atria	heart	E14.5	Tsai lab	GSE46497	4359
DHSs	Dnase1 hypersensitivity	mm9	heart	heart	adult	ENCODE	GSE37074	150000
Gata4	ChIP-seq	mm9	heart	heart	adult	Christoffels lab	GSE35151	150493
Gata4	ChIP-seq	mm9	heart	heart	E12.5	Pu lab	GSE52123	150033
Gata4	ChIP-seq	mm9	heart	heart	adult	Pu lab	GSE52123	150054
Gata4	ChIP-exo	mm9	myocyte cell line	heart	differentiated	Bruneau lab	GSE77548	25710
Gata4	ChIP-exo	mm9	myocyte cell line	heart	cardiac_precursor	Bruneau lab	GSE77548	52999
H3ac	ChIP-seq	mm9	left ventric~~	heart	adult	Baker lab	GSE63590	150201
H3K27ac	ChIP-seq	mm9	avc	heart	E10.5	Christoffels lab	GSE55611	87572
H3K27ac	ChIP-seq	mm9	right atrium	heart	E10.5	Christoffels lab	GSE55611	67092
H3K27ac	ChIP-seq	mm9	heart	heart	adult	ENCODE	GSE49847	53034
H3K27ac	ChIP-seq	mm9	heart	heart	E14.5	ENCODE	GSE49847	84442
H3K27ac	ChIP-seq	mm9	heart	heart	adult	Pennacchio lab	GSE52386	150515
H3K27ac	ChIP-seq	mm9	heart	heart	E11.5	Pennacchio lab	GSE52386	32289
H3K27ac	ChIP-seq	mm9	heart	heart	E14.5	Pennacchio lab	GSE52386	31815
H3K27ac	ChIP-seq	mm9	heart	heart	E17.5	Pennacchio lab	GSE52386	23210
H3K27ac	ChIP-seq	mm9	heart	heart	P0	Pennacchio lab	GSE52386	39133
H3K27ac	ChIP-seq	mm9	heart	heart	P7	Pennacchio lab	GSE52386	36447
H3K27ac	ChIP-seq	mm9	heart	heart	P21	Pennacchio lab	GSE52386	7376
H3K27me3	ChIP-seq	mm9	heart	heart	adult	ENCODE	GSE49847	21876
H3K4me1	ChIP-seq	mm9	heart	heart	adult	ENCODE	GSE49847	150914
H3K4me3	ChIP-seq	mm9	heart	heart	adult	ENCODE	GSE49847	53034
Hand2 (rep1)	ChIP-seq	mm9	heart	heart	adult	Zeller lab	confidential?	150002
Hand2 (rep2)	ChIP-seq	mm9	heart	heart	adult	Zeller lab	confidential?	20446
Hif1a rep1	ChIP-seq	mm9	heart	heart	E12.5	Keck institute	GSE61247	17289
Hopx	ChIP-seq	mm9	heart	heart	E9.5	Won lab	GSE67251	3774
Hopx with EGS	ChIP-seq	mm9	heart	heart	E9.5	Won lab	GSE67251	178
Isl1	ChIP-seq	mm9	sinus node	heart	p3	Evans lab	GSE68974	2322
Mef2a	ChIP-exo	mm9	C2C12 cell line	heart	myoblast	McDermott lab	GSE61204	2796
Nkx2-5	ChIP-seq	mm9	heart	heart	adult	Christoffels lab	GSE35151	150227
Nkx2-5	ChIP-seq	mm9	heart	heart	E12.5	Chen lab	GSE70332	3967
Nkx2-5	ChIP-exo	mm9	myocyte cell line	heart	differentiated	Bruneau lab	GSE77548	62218
Nkx2-5	ChIP-exo	mm9	myocyte cell line	heart	cardiac_precursor	Bruneau lab	GSE77548	9463
p300 (rep1)	ChIP-seq	mm9	heart	heart	adult	ENCODE	GSE49847	137325
p300 (rep2)	ChIP-seq	mm9	heart	heart	adult	ENCODE	GSE49847	116158
Pol2	ChIP-seq	mm9	heart	heart	adult	ENCODE	GSE49847	58637
RBFox2	ChIP-seq	mm9	heart	heart	9w	Palade lab	GSE57926	151144
Shox2	ChIP-seq	mm9	heart	heart	E12.5	Chen lab	GSE70332	x
Smarca4	ChIP-seq	mm9	heart	heart	E11.5	Pennacchio lab	GSE37151	13775
SOX9	ChIP-seq	mm9	AVC~~	heart	E12.5	Fox lab	GSE73225	x
ß-catenin biotin	ChIP-seq	mm9	mESC	heart	blastocyst	McMahon lab	GSE43565	20408
ß-catenin flag	ChIP-seq	mm9	mESC	heart	blastocyst	McMahon lab	GSE43565	20294
Suz12	ChIP-seq	mm9	heart	heart	9w	Palade lab	GSE57926	22476
Tbx20	ChIP-seq	mm9	heart	heart	adult	Evans lab	GSE30943	4011
TBX3	ChIP-seq	mm9	heart	heart	adult	Christoffels lab	GSE44821	150859
Tbx5	ChIP-seq	mm9	HL-1 cells	heart	cell line	Pu lab	GSE21529	48221
Tbx5	ChIP-exo	mm9	myocyte cell line~	heart	differentiated	Bruneau lab	GSE77548	45540
Tbx5	ChIP-exo	mm9	myocyte cell line~	heart	cardiac_precursor	Bruneau lab	GSE77548	8587
Tcf3	ChIP-seq	mm9	mESC	heart	blastocyst	Whitehead Inst.	GSE11724	7336
Smarca4	ChIP-seq	mm9	limb	limb	E11.5	Pennacchio lab	GSE37151	5251
SOX9	ChIP-seq	mm9	Limb	limb	E12.5	Fox lab	GSE73225	151213
ATAC	ATAC-seq	mm9	liver	liver	E12.5	Christoffels lab	in house	70760
Hey2	ChIP-seq	mm9	mESC	stem cell	blastocyst	Gessler	TBA	99750
H3K27ac	ChIP-seq	mm9	forebrain	brain	adult	Pennacchio lab	GSE52386	33178
H3K27ac	ChIP-seq	mm9	forebrain	brain	E11.5	Pennacchio lab	GSE52386	15823
H3K27ac	ChIP-seq	mm9	forebrain	brain	E14.5	Pennacchio lab	GSE52386	27288
H3K27ac	ChIP-seq	mm9	forebrain	brain	E17.5	Pennacchio lab	GSE52386	20450
H3K27ac	ChIP-seq	mm9	forebrain	brain	P0	Pennacchio lab	GSE52386	30308
H3K27ac	ChIP-seq	mm9	forebrain	brain	P7	Pennacchio lab	GSE52386	36771
H3K27ac	ChIP-seq	mm9	forebrain	brain	P21	Pennacchio lab	GSE52386	15246
H3K27ac	ChIP-seq	mm9	forelimb	limb	E11.5	Menke lab	GSE64055	40303
H3K27ac	ChIP-seq	mm9	hindlimb	limb	E11.5	Menke lab	GSE64055	35166
Pol2	ChIP-seq	mm9	limb	limb	E14.5	ENCODE	GSE49847	14426
H3K4me1	ChIP-seq	mm9	limb	limb	E14.5	ENCODE	GSE49847	150360
H3K4me3	ChIP-seq	mm9	limb	limb	E14.5	ENCODE	GSE49847	19259
H3K27ac	ChIP-seq	mm9	limb	limb	E14.5	ENCODE	GSE49848	30543
DHSs	Dnase1 hypersensitivity	mm9	forelimb	limb	E11.5	ENCODE	GSE37074	151669
DHSs	Dnase1 hypersensitivity	mm9	hindlimb	limb	E11.5	ENCODE	GSE37074	150814
H3K27ac	ChIP-seq	mm9	forebrain	brain	E12.5	Rockowitz	GSE66961	26863
H3K4me1	ChIP-seq	mm9	forebrain	brain	E12.5	Rockowitz	GSE66961	40773
H3K4me3	ChIP-seq	mm9	forebrain	brain	E12.5	Rockowitz	GSE66961	15407
H3K27me3	ChIP-seq	mm9	forebrain	brain	E12.5	Rockowitz	GSE66961	25473
Pol2	ChIP-seq	mm9	forebrain	brain	E12.5	Rockowitz	GSE66961	1704
H3K4me1	ChIP-seq	mm9	midbrain	brain	E11.5	Endo	GSE49652	19384
H3K27ac	ChIP-seq	mm9	midbrain	brain	E11.5	Endo	GSE49652	22752
p300	ChIP-seq	mm9	midbrain	brain	E11.5	Pennacchio lab	GSE22549	2786
p300	ChIP-seq	mm9	heart	heart	E11.5	Pennacchio lab	GSE22549	3597
p300	ChIP-seq	mm9	forebrain	brain	E11.5	Pennacchio lab	GSE13845	2454
p300	ChIP-seq	mm9	midbrain	brain	E11.5	Pennacchio lab	GSE13845	562
p300	ChIP-seq	mm9	limb	limb	E11.5	Pennacchio lab	GSE13845	2106
DHSs	Dnase1 hypersensitivity	mm9	cerebellum	brain	P7	Crawford	GSE60731	100000
DHSs	Dnase1 hypersensitivity	mm9	cerebellum	brain	P14	Crawford	GSE60731	100000
DHSs	Dnase1 hypersensitivity	mm9	cerebellum	brain	Adult	Crawford	GSE60731	100000
DHSs	Dnase1 hypersensitivity	mm9	heart	heart	Adult	Crawford	GSE60731	100000
Zic	ChIP-seq	mm9	cerebellum	brain	P7	Crawford	GSE60731	53761
Zic	ChIP-seq	mm9	cerebellum	brain	adult	Crawford	GSE60731	47573
H3K27ac	ChIP-seq	mm9	cerebellum	brain	adult	Creyghton	GSE67978	35904
Smarca4	ChIP-seq	mm9	hindbrain	brain	E11.5	Pennacchio lab	GSE37151	5461
Ncoa1	ChIP-seq	mm9	hindbrain	brain	E11.5	Pennacchio lab	GSE37151	1415
Ncoa2	ChIP-seq	mm9	hindbrain	brain	E11.5	Pennacchio lab	GSE37151	3445
Smarca4	ChIP-seq	mm9	midbrain	brain	E11.5	Pennacchio lab	GSE37151	1415
Smarcc2	ChIP-seq	mm9	hindbrain	brain	E11.5	Pennacchio lab	GSE37151	2273
H3K4me1	ChIP-seq	mm9	limb	limb	E11.5	Pennacchio lab	GSE37151	58181
H3K4me3	ChIP-seq	mm9	limb	limb	E11.5	Pennacchio lab	GSE37151	13137
H3K27me3	ChIP-seq	mm9	limb	limb	E11.5	Pennacchio lab	GSE37151	19668
H3K27ac	ChIP-seq	mm9	limb	limb	E11.5	Pennacchio lab	GSE37151	17118
H3K4me1	ChIP-seq	mm9	brain	forebrain	E11.5	Pennacchio lab	GSE37151	34848
H3K4me3	ChIP-seq	mm9	brain	forebrain	E11.5	Pennacchio lab	GSE37151	16544
H3K27me3	ChIP-seq	mm9	brain	forebrain	E11.5	Pennacchio lab	GSE37151	11073

Supplemental Table 1. a Mouse functional genomic datasets included in merge as possible predictors

Dataset	Type	Genome	Tissue	Stage	Source	Deposit	# of Peaks
Conservation	PhastCons	hg19	all	all	Siepel lab	UCSC	10121233 (174584)*
CTCF	ChIP-seq	hg19	HCM cells	cell line	ENCODE	GSE30263	41966
DHSs	Dnase1 hypersensitivity	hg19	cerebellum	adult	ENCODE	GSE32970	104575
DHSs	Dnase1 hypersensitivity	hg19	cerebrum	adult	ENCODE	GSE32970	135930
DHSs	Dnase1 hypersensitivity	hg19	frontal_cortex	adult	ENCODE	GSE32970	110989
DHSs	Dnase1 hypersensitivity	hg19	HCM cells	cell line	ENCODE	GSE26328	317895
DHSs	Dnase1 hypersensitivity	hg19	heart	adult	ENCODE	GSE32970	124949
H3K27ac	ChIP-seq	hg19	brain	fetal 12w post-gest.	Guo lab	GSE63634	77355
H3K27ac	ChIP-seq	hg19	cerebellum	adult	Creyghton lab	GSE40465	174090
H3K27ac	ChIP-seq	hg19	fibroblast cell line	induced myocytes D6	Cao lab	GSE78096	33954
H3K27ac	ChIP-seq	hg19	fibroblast cell line	induced myocytes D11	Cao lab	GSE78096	36649
H3K27ac	ChIP-seq	hg19	heart	fetal 12w post-gest.	Guo lab	GSE63634	49185
H3K27ac	ChIP-seq	hg19	iPSC	induced myocytes	Sakabe lab	GSE77267	19528
H3K27ac	ChIP-seq	hg19	left ventricle	adult	Ren lab	GSE16256	59070
H3K27ac	ChIP-seq	hg19	limbs	gest. E33	Cotney lab	GSE42413	72841
H3K27ac	ChIP-seq	hg19	limbs	gest. E41	Cotney lab	GSE42413	42284
H3K27ac	ChIP-seq	hg19	limbs	gest. E44	Cotney lab	GSE42413	69409
H3K27ac	ChIP-seq	hg19	limbs	gest. E47	Cotney lab	GSE42413	54981
H3K27ac	ChIP-seq	hg19	liver	fetal 12w post-gest.	Guo lab	GSE63634	27741
H3K27ac	ChIP-seq	hg19	right ventricle	adult	Ren lab	GSE16256	126802
H3K27me3	ChIP-seq	hg19	brain	fetal 12w post-gest.	Guo lab	GSE63634	50180
H3K27me3	ChIP-seq	hg19	fibroblast cell line	induced myocytes D6	Cao lab	GSE78096	275
H3K27me3	ChIP-seq	hg19	fibroblast cell line	induced myocytes D11	Cao lab	GSE78096	173
H3K27me3	ChIP-seq	hg19	heart	fetal 12w post-gest.	Guo lab	GSE63634	34795
H3K27me3	ChIP-seq	hg19	hESC	induced myocytes 14d	Murry lab	SRR577565	86945
H3K27me3	ChIP-seq	hg19	iPSC	induced myocytes	Sakabe lab	GSE77267	8952
H3K27me3	ChIP-seq	hg19	liver	fetal 12w post-gest.	Guo lab	GSE63634	41712
H3K36me3	ChIP-seq	hg19	hESC	induced myocytes 14d	Murry lab	SRR577574	118126
H3K4me1	ChIP-seq	hg19	brain	fetal 12w post-gest.	Guo lab	GSE63634	58426
H3K4me1	ChIP-seq	hg19	heart	fetal (d105)	BROAD institute	GSE17312	113536
H3K4me1	ChIP-seq	hg19	heart	fetal 12w post-gest.	Guo lab	GSE63634	59026
H3K4me1	ChIP-seq	hg19	iPSC	induced myocytes	Sakabe lab	GSE77267	13902
H3K4me1	ChIP-seq	hg19	liver	fetal 12w post-gest.	Guo lab	GSE63634	43426
H3K4me3	ChIP-seq	hg19	brain	fetal 12w post-gest.	Guo lab	GSE63634	16745
H3K4me3	ChIP-seq	hg19	fibroblast cell line	induced myocytes D6	Cao lab	GSE78096	18720
H3K4me3	ChIP-seq	hg19	fibroblast cell line	induced myocytes D11	Cao lab	GSE78096	19871
H3K4me3	ChIP-seq	hg19	HCM cells	cell line	ENCODE	GSE35583	49247
H3K4me3	ChIP-seq	hg19	heart	fetal (d105)	BROAD institute	GSE17312	1238
H3K4me3	ChIP-seq	hg19	heart	fetal 12w post-gest.	Guo lab	GSE63634	86937
H3K4me3	ChIP-seq	hg19	hESC	induced myocytes 14d	Murry lab	SRR577568	26960
H3K4me3	ChIP-seq	hg19	iPSC	induced myocytes	Sakabe lab	GSE77267	10760
H3K4me3	ChIP-seq	hg19	liver	fetal 12w post-gest.	Guo lab	GSE63634	11799
H3K9ac	ChIP-seq	hg19	heart	fetal (d105)	BROAD institute	GSE17312	49876
p300	ChIP-seq	hg19	cortex	fetal (gest. week 20)	Pennacchio lab	GSE42881	642876
p300	ChIP-seq	hg19	heart	adult	Pennacchio lab	GSE32587	139824
p300	ChIP-seq	hg19	heart	fetal (gest. week 16)	Pennacchio lab	GSE32587	108384
Pol2	ChIP-seq	hg19	heart	fetal (gest. week 16)	Pennacchio lab	GSE32587	223086
Pol2	ChIP-seq	hg19	iPSC	induced myocytes	Sakabe lab	GSE77267	11332
ATAC	ATAC-seq	hg19	SANLPCs	induced SAN myocytes	Protze lab	unpublished	45389
ATAC	ATAC-seq	hg19	hESC	0d H1 differentiated myocytes	Snyder lab	GSE85330	54378
ATAC	ATAC-seq	hg19	hESC	2d H1 differentiated myocytes	Snyder lab	GSE85330	21979
ATAC	ATAC-seq	hg19	hESC	4d H1 differentiated myocytes	Snyder lab	GSE85330	28991
ATAC	ATAC-seq	hg19	hESC	30d H1 differentiated myocytes	Snyder lab	GSE85330	11931
ATAC	ATAC-seq	hg19	hESC	0d H9 differentiated myocytes	Snyder lab	GSE85330	28417
ATAC	ATAC-seq	hg19	hESC	2d H9 differentiated myocytes	Snyder lab	GSE85330	10980
ATAC	ATAC-seq	hg19	hESC	4d H9 differentiated myocytes	Snyder lab	GSE85330	26580
ATAC	ATAC-seq	hg19	hESC	30d H9 differentiated myocytes	Snyder lab	GSE85330	24661
ATAC	ATAC-seq	hg19	iPSC	0d C15 induced myocytes	Snyder lab	GSE85330	37100
ATAC	ATAC-seq	hg19	iPSC	2d C15 induced myocytes	Snyder lab	GSE85330	35192
ATAC	ATAC-seq	hg19	iPSC	4d C15 induced myocytes	Snyder lab	GSE85330	13920
ATAC	ATAC-seq	hg19	iPSC	30d C15 induced myocytes	Snyder lab	GSE85330	16191
ATAC	ATAC-seq	hg19	iPSC	0d C20 induced myocytes	Snyder lab	GSE85330	27217
ATAC	ATAC-seq	hg19	iPSC	2d C20 induced myocytes	Snyder lab	GSE85330	22065
ATAC	ATAC-seq	hg19	iPSC	4d C20 induced myocytes	Snyder lab	GSE85330	51994
ATAC	ATAC-seq	hg19	iPSC	30d C20 induced myocytes	Snyder lab	GSE85330	19602
ATAC	ATAC-seq	hg19	hESC	cardiac mesoderm H7	Weissman lab	GSE85066	180380
ATAC	ATAC-seq	hg19	hiPSC-derived VLCM	induced LV myocytes	Protze lab	unpublished	38335
ATAC	ATAC-seq	hg19	left atrium	adult	Martin lab	unpublished	55045
DHSs	Dnase1 hypersensitivity	hg19	limbs	fetal	ENCODE	GSE90405	95652
Med1	ChIP-seq	hg19	iPSC	induced myocytes 32d	Srivastava lab	GSE85631	15400

*number of ultra-conserved phastCons (score≥600)

Supplemental Table 1. b Human functional genomic datasets included in merge as possible predictors

	Scn5a$^{\Delta 7}$ at E10.5	ΔRE6-8 at E13.5	ΔRE6-9 at E10.5	ΔRE6-9 at E13.5	ΔRE6-9 at p7
WT	6	13	16	13	6
Heterozygotes	24	27	41	29	12
Homozygotes	8	12	12	13	0
χ2	2.842	0.115	3.909	0.164	6.000
P	0.241	0.994	0.142	0.921	0.050

	Scn5a$^{\Delta 7/\Delta RE6\text{-}9}$ (A) at E10.5	*Scn5a*$^{\Delta 7/\Delta RE6\text{-}9}$ (B) at E10.5
WT	24	19
Heterozygotes	40	40
Homozygotes	0	0
χ2	22.000	19.712
P	<0.050	<0.050

Supplementary Table 2. Overview of mice born from heterozygote crosses.

TALEN	Genomic location (mm9)	RVD sequence
TAL1	chr9: 119469416 - 119469431	HD NI NH HD HD HD HD NG NG HD HD NI HD HD HD
TAL2	chr9: 119469385 - 119469400	NI HD HD NI NI NI NI NG HD NG NG HD NI HD NI
TAL3	chr9: 119374922 - 119374937	NI NH HD NG NH NI HD NH HD NG NG NH NG NH NH
TAL4	chr9: 119374891 - 119374907	NH NI NH NG NG HD HD NG HD NG NI NH NH NH NH NH NG HD
TAL5	chr9: 119392340 - 119392359	NH NH NH NI NI NG NH NI HD NG NG HD NI NH NH NG NG NH NG HD
TAL6	chr9: 119392305 - 119392324	NH HD HD NG NH NI NH HD HD NG HD NG NH HD NG NG HD HD NH NH
TAL7	chr9: 119388720 - 119388748	NH HD HD NI HD HD HD NI NH NI HD HD HD HD NI NH HD HD NG NI NI HD HD HD HD HD NI NG NG NG
TAL8	chr9: 119388765 - 119388785	NG NG NH NG NI HD HD NG NI NI NG NI NI NI NI NG NH NG HD NI

CRISPR	Genomic location (mm9)	Sequence
CRISPR1	chr9: 119377940	GGTCTGAGTACCGTAGATGA
CRISPR2	chr9: 119380243	GGCACTATTTGAGTTCCACT
CRISPR3	chr9: 119390183	GGGAGCCGAGGGCGCTCCTT

Supplementary Table 3. TALEN location and RVD sequences, and CRISPR location and sequences for all transgenic mouse models.

qPCR primers	Sequence 5' to 3'
Scn5a	Forward: GGGACTCATTGCCTACATGA
	Reverse: GCACTGGGAGGTTATCACTG
Scn10a	Forward: CTAGTCTGTTGTTTTCTGCG
	Reverse: GCGAAGAGCAGCGTGCGAATC
Exog	Forward: GTCGTGAGCTGACGGAGAGAT
	Reverse: CGAGCCAGGATCACCTTGTAAA
Xylb	Forward: CGGGAGGAGCATCTCACAATAA
	Reverse: CATGAAAAGCTCGGTATGCGGA
Acaa1a	Forward: TGTGGCTGAGCGGTTTGGCATT
	Reverse: TGTCACCCTTGTCATCCAGGAC
Acaa1b	Forward: CCTTGGGGTCCTGAGGTCCTAT
	Reverse: CCTAACTTCTCCACACAGTAGA
Myd88	Forward: CCGCATGGTGGTGGTTGTTTCT
	Reverse: GGAATCAGTCGCTTCTGTTGGA
Oxsr1	Forward: GAGAGATACAGCAGAGGGTGTT
	Reverse: CACCAGATGCCAGTTTGAAAGT
Osr1	Forward: CACACTGATGAGCGACCTTACA
	Reverse: CCCACACTCTTGACACTTGAAA
Slc22a13	Forward: AGACCCTGAAGGACACCCTCCA
	Reverse: CACCCTCACACTGGAAGTTCTT
Slc22a14	Forward: CTCCGTGACAGCATAGAGTACA
	Reverse: CACTTCAGAATCAGCCACAATG
Dlec1	Forward: AGCCAGGGAGGATCATGCGTTT
	Reverse: CCACCACCAGTGTAGATTCATA
Plcd1	Forward: GCCGTCAGACAGCAGTTATTAC
	Reverse: AGGAGTCATAATCCTCTACCAT
Vill	Forward: ACAGCCAGTCCTACGAGGAGAT
	Reverse: GGTGACCTGCCTTGTTATCACT
Acvr2b	Forward: TGGACATCCATGAGGACCCG
	Reverse: GTTCACTCTGCCACGACTGC
Wdr48	Forward: ATGAGAGTGACTGCTTCGCT
	Reverse: CCCCACTCACATGGTTCACTT
Scn11a	Forward: GCCAGCCCAACGAAGTGAA
	Reverse: TGTTCTCCAAGCCAGAAACCA
Gorasp1	Forward: TTGGGTTCCAGGCAGAGTGA
	Reverse: GGGCACACGCTTATGTTGC
cTnI	Forward: TCGGGTGGACAAAGTGGATG
	Reverse: AAACTTGCCACGGAGGTCAT
Hprt	Forward: TGTTGGATATGCCCTTGACT
	Reverse: GATTCAACTTGCGCTCATCT
Eef1e1	Forward: TCCAGTAAAGAAGACACCCAG
	Reverse: GACAAAACCAGCGAGACACA
Tnnt2	Forward: TTCGACCTGCAGGAAAAGTT
	Reverse: CTTCCCACGAGTTTTGGAGA

Supplementary Table 4 qPCR primers sequences.

vpname	nReads	nMapped	nMappedCis	nMappedCisperc	Reads	cisReads	percCis	cisReads.nTop	percCis.nTop	Captures	cisCaptures	topPct	capt100Kb	cov100Kb	capt1Mb	cov1Mb
Scn5a_P1_2_WT1	923567	850745	489669	57.56	733994	471819	64.28	216403	45.37	34737	10762	54.13	71.26	40.37	33.16	61.36
Scn5a_P1_2_WT2	800213	743394	523278	70.39	682917	515267	75.45	110269	40.02	28436	8722	78.6	65.2	44.66	28.68	64.83
Scn5a_P1_2_WT3	421983	380629	218668	57.45	330503	211534	64	90059	43.27	26733	8295	57.43	59.36	40.51	25.49	60.49
Scn5a_P1_2_del1	1175210	1083775	742011	68.47	938381	703689	74.99	463086	66.46	52110	26363	34.19	74.67	32.45	47.76	52.16
Scn5a_P1_2_del2	981222	903112	621741	68.84	788718	592060	75.07	372880	65.59	45283	23473	37.02	75.66	32.38	46.01	52.24
Scn5a_P1_2_del3	732917	674346	460840	68.34	585410	438292	74.87	284416	66.03	41178	21597	35.11	71.48	32.69	43.8	52.3
Scn5a_P1_2_liver1	720128	656658	415523	63.28	584293	406338	69.54	112229	38.94	43804	8594	72.38	66.41	43.05	26.82	64.27
Scn5a_P1_2_liver2	847913	771881	455322	58.99	669697	440749	65.81	178922	44.06	43053	11193	59.41	70.04	39.43	32.71	61.39
Scn5a_P1_2_liver3	896322	824211	492942	59.81	719216	477303	66.36	185101	43.48	44689	12986	61.22	72.8	40.1	35.41	61.78
Scn5a_enh_WT1	558262	512843	224611	43.8	404797	206939	51.12	201786	50.84	31561	10427	2.49	80.49	47.76	32.67	62.46
Scn5a_enh_WT2	284442	260897	117662	45.1	204559	108739	53.16	104156	52.45	25732	8563	4.21	73.8	51.46	27.91	65.04
Scn5a_enh_WT3	136891	123426	53126	43.04	95209	48710	51.16	46860	50.31	20062	6620	3.8	63.1	50.72	22.43	64.06
Scn5a_enh_del1	690824	637769	411998	64.6	524911	382411	72.85	374671	72.56	48604	27123	2.02	81.94	38.34	49.34	53.26
Scn5a_enh_del2	785692	726629	470061	64.69	598942	436597	72.89	426611	72.54	42852	24087	2.29	79.26	38.82	47.71	53.73
Scn5a_enh_del3	749894	689277	440080	63.85	563553	406920	72.21	399145	71.93	41748	23312	1.91	81.49	37.88	47.6	52.91
Scn5a_enh_liver1	259380	237014	104648	44.15	188262	96557	51.29	91533	50.14	21734	7128	5.2	71.91	50.49	26.06	65.83
Scn5a_enh_liver2	554362	510804	222341	43.53	400269	204179	51.01	198184	50.37	31295	10558	2.94	77.15	45.62	32.64	61.87
Scn5a_enh_liver3	450943	413532	177470	42.92	326340	163957	50.24	159266	49.82	36693	12039	2.86	79.49	45.32	35.18	60.97

Supplementary Table 5. Stats report on 4C-seq analysis of whole hearts from 8-week old heterozogous RE6-9 and wildtype mice, and liver from 8-week old wildtype mice.

References

1. Visel A, Minovitsky S, Dubchak I, Pennacchio LA. VISTA Enhancer Browser--a database of tissue-specific human enhancers. *Nucleic Acids Res* **35**, D88-92 (2007).

2. van Duijvenboden K, de Boer BA, Capon N, Ruijter JM, Christoffels VM. EMERGE: a flexible modelling framework to predict genomic regulatory elements from genomic signatures. *Nucleic Acids Res* **44**, (2015).
