# Supplementary material for: An enhancer cluster controls gene activity and topology of the SCN5A-SCN10A locus in vivo
Source: Nat Commun. 2019 Oct 30;10:4943. doi: 10.1038/s41467-019-12856-5 (PMC6821807; doi:10.1038/s41467-019-12856-5)
Supplement: Supplementary file 3 — Reporting Summary [file 41467_2019_12856_MOESM3_ESM.pdf]

nature research

Corresponding author(s): Vincent M. Christoffels

Last updated by author(s): Sep 15, 2019

Reporting Summary

Nature Research wishes to improve the reproducibility of the work that we publish. This form provides structure for consistency and transparency in reporting. For further information on Nature Research policies, see Authors & Referees and the Editorial Policy Checklist.

Statistics

For all statistical analyses, confirm that the following items are present in the figure legend, table legend, main text, or Methods section.

n/a	Confirmed	
☐	☒	The exact sample size (*n*) for each experimental group/condition, given as a discrete number and unit of measurement
☐	☒	A statement on whether measurements were taken from distinct samples or whether the same sample was measured repeatedly
☐	☐	The statistical test(s) used AND whether they are one- or two-sided<br>*Only common tests should be described solely by name; describe more complex techniques in the Methods section.*
☒	☐	A description of all covariates tested
☒	☐	A description of any assumptions or corrections, such as tests of normality and adjustment for multiple comparisons
☐	☒	A full description of the statistical parameters including central tendency (e.g. means) or other basic estimates (e.g. regression coefficient) AND variation (e.g. standard deviation) or associated estimates of uncertainty (e.g. confidence intervals)
☐	☒	For null hypothesis testing, the test statistic (e.g. *F*, *t*, *r*) with confidence intervals, effect sizes, degrees of freedom and *P* value noted<br>*Give P values as exact values whenever suitable.*
☒	☐	For Bayesian analysis, information on the choice of priors and Markov chain Monte Carlo settings
☒	☐	For hierarchical and complex designs, identification of the appropriate level for tests and full reporting of outcomes
☒	☐	Estimates of effect sizes (e.g. Cohen's *d*, Pearson's *r*), indicating how they were calculated

Our web collection on statistics for biologists contains articles on many of the points above.

Software and code

Policy information about availability of computer code

Data collection	No software was used for data collection
Data analysis	Galaxy (https://usegalaxy.org/ ;Galaxy version 19.01.rc1) was used for the joining of files based on either genome coordinates or gene name (EMSEMBLE Biomart).<br>EMERGE program published (van Duijvenboden, K. et al. EMERGE: a flexible modelling framework to predict genomic regulatory elements from genomic signatures. Nucleic Acids Res. 2016 Mar 18;44(5)e42. doi: 10.1093/nar/gkv1144).

For manuscripts utilizing custom algorithms or software that are central to the research but not yet described in published literature, software must be made available to editors/reviewers. We strongly encourage code deposition in a community repository (e.g. GitHub). See the Nature Research guidelines for submitting code & software for further information.

Data

Policy information about availability of data

All manuscripts must include a data availability statement. This statement should provide the following information, where applicable:

- Accession codes, unique identifiers, or web links for publicly available datasets
- A list of figures that have associated raw data
- A description of any restrictions on data availability

RNA-seq data and 4C data that supports the findings of this study have been deposited in NCBI's Gene Expression Omnibus (Edgar et al., 2002) and are accessible through GEO Series accession number GSE129067 (https://www.ncbi.nlm.nih.gov/geo/query/acc.cgi?acc=GSE129067).

To review GEO accession GSE129067:
Go to https://www.ncbi.nlm.nih.gov/geo/query/acc.cgi?acc=GSE129067
Enter token ktinkcqobxuztqt into the box

Field-specific reporting

Please select the one below that is the best fit for your research. If you are not sure, read the appropriate sections before making your selection.

☒ Life sciences ☐ Behavioural & social sciences ☐ Ecological, evolutionary & environmental sciences

For a reference copy of the document with all sections, see nature.com/documents/nr-reporting-summary-flat.pdf

Life sciences study design

All studies must disclose on these points even when the disclosure is negative.

Sample size	No sample-size calculation was performed in this study. For initial analysis we start of with a group size of n=3 and we continue with an in-dept analysis (e.g. qPCR and ECG analyses) by increasing the group size as described in this study to observe differences between groups. For RNA-seq and 4C we based our sample size on previous performed studies where a group size of n=3 was sufficient for these analyses.
Data exclusions	No data were excluded from the analyses.
Replication	All attempts at replication were successfull (e.g. 4C, RNA-seq, qPCR).
Randomization	Randomization not relevant to this study. Mice were selected based on genotype.
Blinding	Investigators involved in ECG recordings and optical mapping, and analyses were blinded, and were not aware of the genotype of laboratory animals (mice). After ECG recordings and analyses the laboratory animals were checked for their genotype.

Reporting for specific materials, systems and methods

We require information from authors about some types of materials, experimental systems and methods used in many studies. Here, indicate whether each material, system or method listed is relevant to your study. If you are not sure if a list item applies to your research, read the appropriate section before selecting a response.

Materials & experimental systems

n/a	Involved in the study	
☐	☒	Antibodies
☐	☒	Eukaryotic cell lines
☒	☐	Palaeontology
☐	☒	Animals and other organisms
☒	☐	Human research participants
☒	☐	Clinical data

Methods

n/a	Involved in the study	
☒	☐	ChIP-seq
☒	☐	Flow cytometry
☒	☐	MRI-based neuroimaging

Antibodies

Antibodies used	Nav1.5 antibody rabbit polyclonal (SKM2, SCN5A); Sigma-Aldrich; catalog number: S0819<br>Calnexin mouse monoclonal (Clone C8.B6); Millipore; MAB3126
Validation	Nav 1.5:<br>- Manufacturer's website https://www.sigmaaldrich.com/catalog/product/sigma/s0819?lang=en&region=NL<br>- Data provided in manuscript<br><br>Calnexin:<br>- Manufacturer's website http://www.merckmillipore.com/NL/en/product/Anti-Calnexin-Antibody-ER-marker-clone-C8.B6,MM_NF-MAB3126?ReferrerURL=https%3A%2F%2Fwww.google.nl%2F&bd=1<br>- Data provided in manuscript

Eukaryotic cell lines

Policy information about cell lines

Cell line source(s)	H10
Authentication	H10 (RRID:CVCL_W080)
Mycoplasma contamination	All cell lines tested negative for mycoplasma contamination.

Commonly misidentified lines (See ICLAC register)	Not applicable

Animals and other organisms

Policy information about studies involving animals; ARRIVE guidelines recommended for reporting animal research

Laboratory animals	Mouse; FVB/NRj; sexes: males and females; age: 0 - 3 months; embryonic day: E10.5 and E13.5<br>Mouse; SPRET/Eij; sexes: males and females; age: 2 weeks
Wild animals	The study did not involve wild animals.
Field-collected samples	The study did not involve samples collected from the field.
Ethics oversight	Animal care and experiments conform to the Directive 2010/63/EU of the European Parliament. All animal work was approved by the Animal Experimental Committee of the Academic Medical Center, Amsterdam, and was carried out in compliance with the Dutch government guidelines. All animal experiments were done under DAE285 (IvD, Academic Medical Center, Amsterdam).

Note that full information on the approval of the study protocol must also be provided in the manuscript.
